# Supplementary material for: Mechanisms governing the pioneering and redistribution capabilities of the non-classical pioneer PU.1
Source: Nat Commun. 2020 Jan 21;11:402. doi: 10.1038/s41467-019-13960-2 (PMC6972792; doi:10.1038/s41467-019-13960-2)
Supplement: Supplementary file 7 — Source data [file 41467_2019_13960_MOESM7_ESM.zip › Source_Data/FigureS3/FigureS3J_MotifScanOutput/homerResults/motif57.info.html]

Motif 57

## Information for 19-TAGATGGTATAGC (Motif 57)

A
G
C
T
C
G
T
A
C
A
T
G
C
G
T
A
G
A
C
T
C
T
A
G
C
A
T
G
A
C
G
T
T
C
G
A
A
C
G
T
C
T
G
A
T
A
C
G
A
T
G
C
  
Reverse Opposite:
